# Retrograde Interference in Perceptual Learning of a Peripheral Hyperacuity Task

**DOI:** 10.1371/journal.pone.0024556

**Published:** 2011-09-09

**Authors:** Shao-Chin Hung, Aaron R. Seitz

**Affiliations:** Department of Psychology, University of California Riverside, Riverside, California, United States of America; University of Regensburg, Germany

## Abstract

Consolidation, a process that stabilizes memory trace after initial acquisition, has been studied for over a century. A number of studies have shown that a skill or memory must be consolidated after acquisition so that it becomes resistant to interference from new information. Previous research found that training on a peripheral 3-dot hyperacuity task could retrogradely interfere with earlier training on the same task but with a mirrored stimulus configuration. However, a recent study failed to replicate this finding. Here we address the controversy by replicating both patterns of results, however, under different experimental settings. We find that retrograde interference occurs when eye-movements are tightly controlled, using a gaze-contingent display, where the peripheral stimuli were only presented when subjects maintained fixation. On the other hand, no retrograde interference was found in a group of subjects who performed the task without this fixation control. Our results provide a plausible explanation of why divergent results were found for retrograde interference in perceptual learning on the 3-dot hyperacuity task and confirm that retrograde interference can occur in this type of low-level perceptual learning. Furthermore, our results demonstrate the importance of eye-movement controls in studies of perceptual learning in the peripheral visual field.

## Introduction

Consolidation, a process that stabilizes memory or skills after initial acquisition, has been studied over a century as a central issue in learning and memory [1]. While consolidation involves multiple sub-processes [2], a key aspect of consolidation involves building up a resistance from interference of new learning. This process of stabilization has been studied in learning of word lists [1], motor learning tasks [2], [3], [4], [5], [6], and perceptual learning [7], [8], [9], [10], [11], [12], [13], [14], [15], [16], [17], and across these disciplines it has been observed that practice with two tasks (Task A and then Task B) in close temporal proximity can result in interference from Task B on Task A. Furthermore, a number of studies [1], [2], [3], [4], [5], [6], [7], [17] demonstrate that a temporal interval between the practicing of two tasks can ameliorate this interference. These behavioral investigations, along with neuroscientific research of stabilization at the synaptic level (e.g. l-LTP [18]) have led to broad agreement that initial learning is liable to interference and that stabilization processes can protect learning from later interference.

However, while there is broad agreement that in many tasks interference of learning can occur, and that there exists processes of stabilization, the time-course and mechanisms by which stabilization occurs at a behavioral level are heavily debated. Early, studies of word learning found that stabilization occurred in a period of 6 minutes [1], studies of perceptual learning show that stabilization can occur over an hour in some settings [7], or within a few minutes in others [17], and studies of motor learning show that in some cases stabilization occurs over 4–6 hours [4], and in others 24 hours is not sufficient [5]. These divergent findings bring into question whether there are common mechanisms of stabilization that are involved in different experimental domains, and, in some cases, bring into question the veracity of certain findings.

Indeed, in the case of perceptual learning, a controversy has arisen regarding whether interference of learning occurs in both a retrograde fashion (i.e. between different blocks of trials) and a trial-wise (i.e. rapidly interleaved trials of different types) basis. This has led to two published studies that used qualitatively similar methods and observed divergent results. In the case of Seitz et al [7], disruption of learning for a hyperacuity task occurred if a second session with an opposite offset side was performed immediately after the first training session. Moreover, a one-hour temporal delay of the second session was sufficient to restore learning. This study suggested that visual perceptual learning also requires a stabilization process to consolidate before being resistant to interference by a second stimulus, and that this interference is specific to the location and orientation of the stimuli. However, a recent study by Aberg and Herzog [12] conducted five experiments testing for retrograde interference in a variety of hyperacuity stimulus sets that produced interference on a trial-wise basis. Four of experiments involved line bisection tasks presented at the fovea, and, one of the experiments tested was modeled after Seitz et al [7]. These authors found no retrograde interference in any of their experiments. The divergent findings of Seitz et al [7] and Aberg and Herzog [12] makes it uncertain whether retrograde interference truly occurs in the peripheral 3-dot hyperacuity task.

To address this controversy, we decided to replicate our initial finding of retrograde interference for 3-dot hyperacuity. To improve the validity of our findings, we rewrote the experimental code from scratch and ran the experiment on different equipment, in a different lab, and with a different subject population than was used in Seitz et al [7]. Also, to ensure tight experimental control we ran the experiment with and without an eye-tracker, which was integrated into the program to create a gaze-contingent stimulus presentation that enforced fixation while subjects performed the task. Of note, neither Seitz et al [7] nor Aberg and Herzog [12] employed an eye-tracker, although both studies instructed subjects to maintain fixation during task-performance. The use of the eye-tracker was important in our task where subjects were asked to fixate a central cross while task-relevant stimuli were always presented in the lower-right peripheral visual field. As we discuss below, the use of an eye-tracker can be important in tasks where peripheral targets are presented in a predicable manner.

## Materials and Methods

### Participants

Thirty subjects who were naïve to research purpose participated and received payment for their participation in the experiment. An extra bonus was given based upon good performance to all subjects who completed all 5 sessions. All subjects reported normal (or corrected-to-normal) binocular visual acuity. Informed consent was obtained from all the subjects and the experiments were conducted in accordance with the IRB approved by the Human Research Review Board of University of California, Riverside.

### Apparatus

The stimuli were presented using Psychophysics Toolbox [19], [20] for MATLAB (The MathWorks, Natick, MA) on a Mac mini computer. The stimuli appeared on a 24″ Sony Trinitron CRT monitor with resolution of 1600×1024 pixels and a refresh rate of 100 Hz. A ViewPoint Eye Tracker system running at 220 Hz (USB-220™, Arrington Research ®) and a head positioner including chin rest were used to facilitate the eye fixation at the center throughout the entire experiment. Layout of eye-tracking system was displayed on PC, the Mac and PC computers communicated through a direct, Ethernet line. The eye-tracking system was programmed so that new trials start once when subjects fixate at the center (within a 2 degree radius fixation window) for 300 ms. If an eye-movement outside of this window was detected at any point after the trial started, which was rare due to the rapid stimulus presentation, then that trial was aborted (and excluded from the analysis) and a new trial was initiated.

### Stimuli

**Figure 1:**
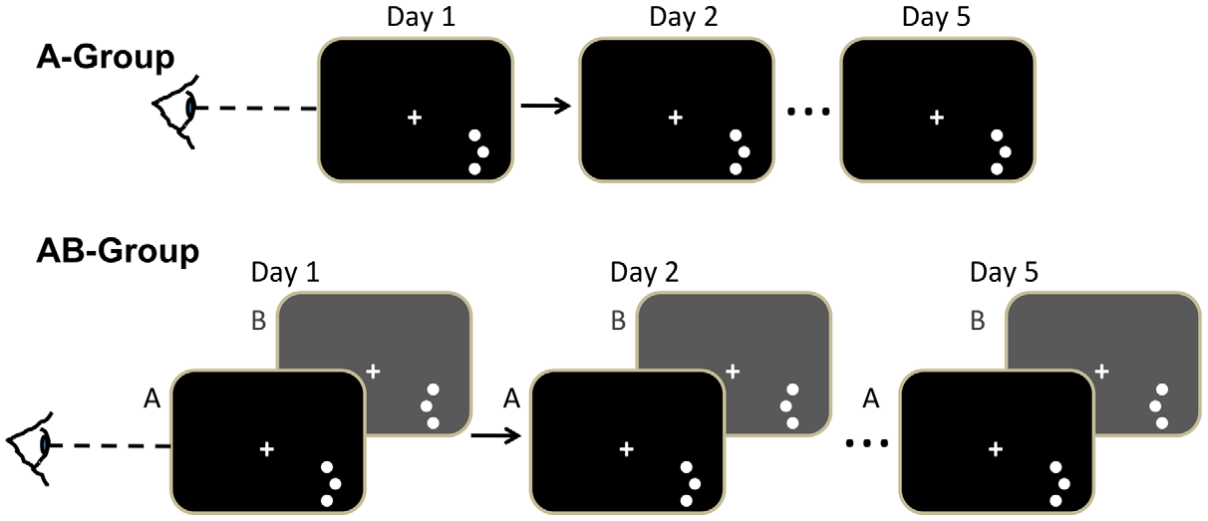
Stimuli and procedure. In the A-group, subjects (n = 12) performed one single session (400 trials) with a single offset side for 5 days. In the AB-group, subjects (n = 12) performed an additional training session B (400 trials) immediately after session A. The three-dot were identical except that the offset side in training session B was opposite to that presented in session A. For both these groups, the eye-tracker was employed with the gaze-contingent display. Note the offset side used in session A and B were counterbalanced across subjects for all experiments. The AB-gazefree group used the same paradigm as for the AB-group except that the eye-tracker was not employed.

The stimuli used were the same as previously reported [7] (see Figure 1). A white, vertical three-dot stimulus was presented on a black background on the monitor. Each dot had a radius of 2′ (arc minute), and the distance between the top and bottom dots was 20′. Each trial consisted of one aligned three-dot stimulus, and one offset stimulus with the middle dot offset to the right or left. We used a set of offset variables representing 5 different difficulties (0.9′, 1.8′, 2.7′, 3.6′, and 4.5′).

### Procedure

Subjects were trained on the three-dot hyperacuity task using the gaze-contingent display that enforced fixation (Figure 1). A central fixation cross was presented on the screen for 300 ms at the beginning of every trial, but to ensure subject's fixation, the stimuli wouldn't appear if subjects didn't fixate at the center. Two stimuli – one aligned and one offset three-dot – were presented successively in the bottom right visual field (7.5° in the periphery). The presentation of each stimulus was 50 ms, separated by an inter-stimulus interval (ISI) of 400 ms. After each trial, subjects had 2 seconds to indicate whether the first stimulus or the second one was offset with a key-press (1 or 2) on the keyboard. Feedback was given as a flash of green cross at the center if the answer was correct, or a flash of red central cross if it was incorrect.

The entire task consisted of 5 training sessions, with each session being conducted at the same time on separate days. The task was typically performed on 5 consecutive days, however in a couple cases there were 1 or 2 days off between sessions. Each training session had 400 trials, divided into 20 blocks (4 blocks per offset size given 5 different offsets), with each block containing 20 trials of the same offset. The order of blocks was randomly mixed in each session, and breaks allowing subjects rest their eyes were given every 5 blocks (every 100 trials).

Subjects were run in one of three conditions. In the A-group (n = 12) subjects conducted 5 sessions on 5 different days in which session (400 trials) involved training with a single offset side. For the AB-group, subjects (n = 12) performed an additional training session B (400 trials) immediately after they completed training session A. Training session B had the same vertical three-dot stimulus as the one in session A, except that the offset side of stimulus in session B was opposite to that presented in session A. Note the offset side used in session A and B were counterbalanced across subjects for all experiments. For both the A-group and the AB-group the eye-tracker was employed with the gaze-contingent display. In the AB-gazefree group, the paradigm was the same as for the AB-group, except that the eye-tracker was not employed.

## Results

**Figure 2:**
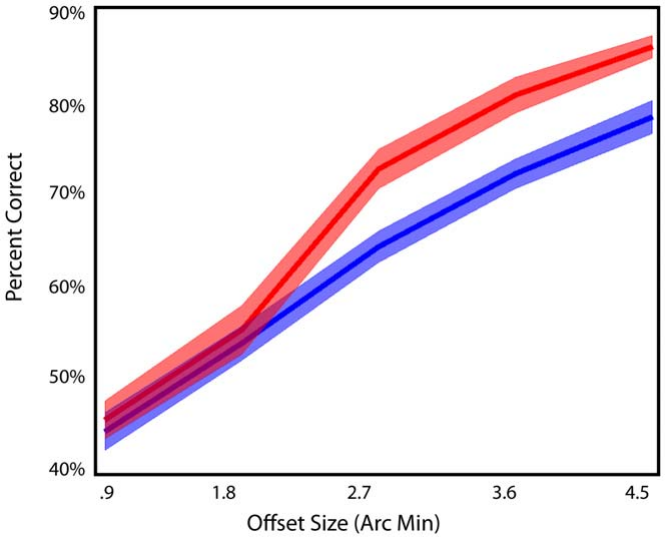
Results from training in the A-group. Pretest (blue) posttest (red). After performing the task with only one offset side for 5 days, subjects showed significant learning that was most prominent in the 2.7′, 3.6′, and 4.5′ offset size conditions. Shaded regions represent standard error.

We first verified that training on a single condition (A) would produce learning. The results from the A-group are shown in Figure 2. Indeed we found significant learning between the first and fifth sessions for this group (F(1,11) = 7.02, p = .023). These results demonstrate that our training procedure is effective.

**Figure 3:**
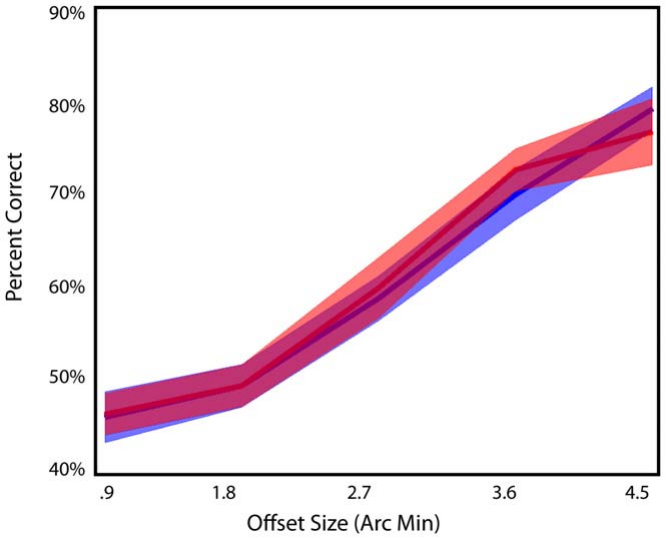
Results from training in the AB-group. Pretest (blue) posttest (red). Subjects performed an additional training session B immediately after session A; offset sides were opposite in session A and B. After 5 days, there was not significant learning found for offset side A. Shaded regions represent standard error.

To verify whether immediate training with a task can interfere a previously learned task, we examined learning for the AB-group. The results for AB-group can be seen in Figure 3. For this group no significant learning was found (F(1,11) = 0.013, p = 0.91). These results replicate our previous finding [7] that retrograde interference can occur in this type of perceptual learning.

**Figure 4:**
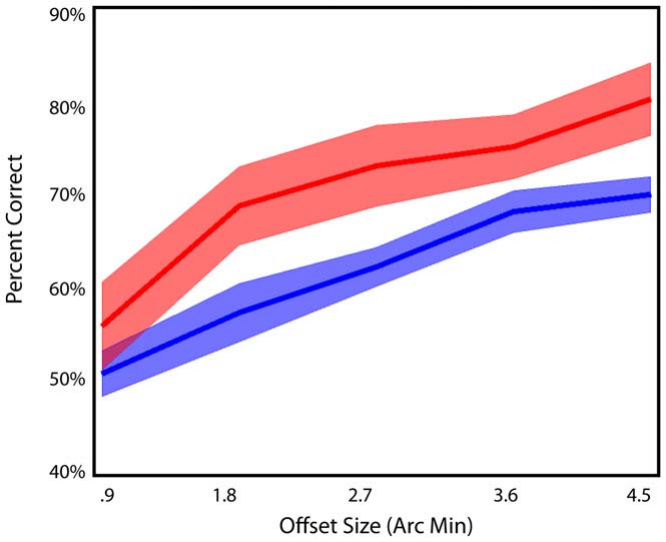
Results from training in the AB-gazefree group. Pretest (blue) posttest (red). Without the use of eye-tracker, subjects showed significant improvement in offset side A after 5 days of AB training. Shaded regions represent standard error.

So far, we've replicated the results of Seitz et al. [7]. To address the controversy raised by Aberg and Herzog [12], another group of subjects was run without eye-tracker. In the AB-gazefree group, six subjects completed five training sessions, just like the AB-group, however without the use of the eye-tracker. It should be noted that subjects were told to fixate at the central cross throughout the experiment with their head positions stabilized with a chin rest. The results in the AB-gazefree group are shown in Figure 4. Significant learning was found when performance was compared between day 5 and day 1 (F(1,5) = 6.93, p = .046). While these results showed significant learning, we found that at least some subjects did not consistently maintain fixation in later sessions (this is a particular problem in a condition where the location of the stimulus is predictable as it was in this study). These results are comparable to those of Aberg and Herzog [12], who claim that perceptual learning does not suffer from retrograde interference of task B on task A either in visual hyperacuity task or bisection task.

**Figure 5:**
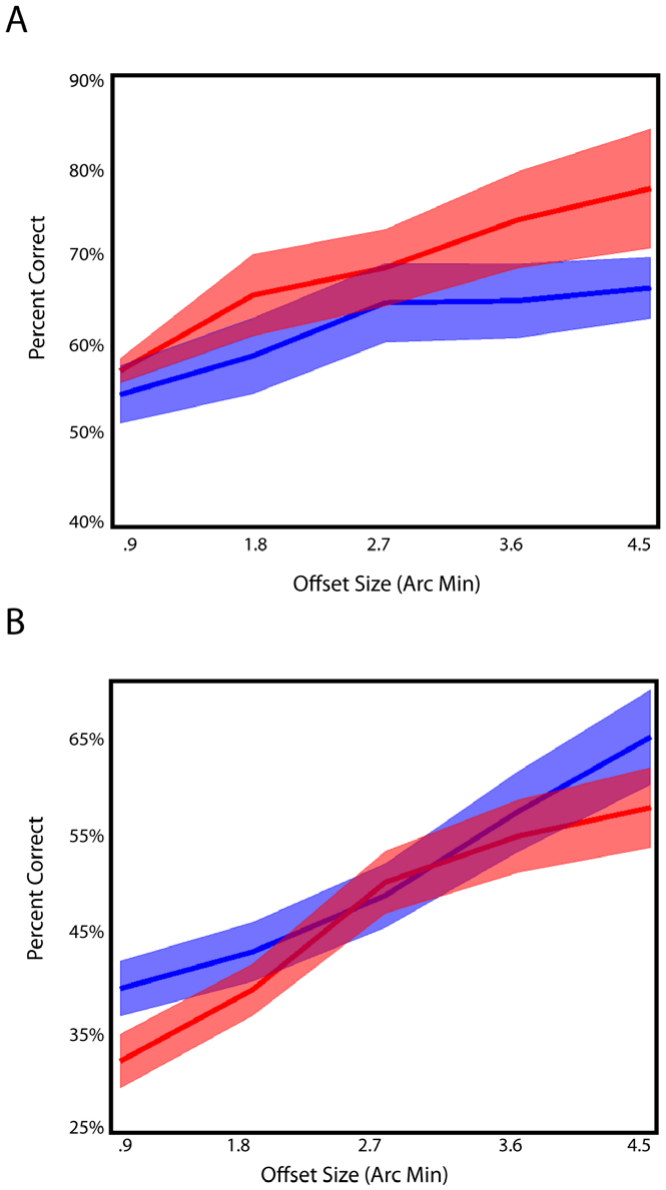
Results from the B condition. A, data from the AB-gazefree group. B, data from the AB group. Pretest (blue) posttest (red). Shaded regions represent standard error.

We also examined performance in the B condition for the AB-gazefree group (Figure 5a) and the AB-group (Figure 5b). No significant learning was found for the B condition in the AB-gazefree group (F(1,5) = .89, p = .39), nor for the B condition in the AB-group F(1,11) = 0.56, p = 0.47). The poor performance in the B group was also observed in Seitz et al. [7] and may simply reflect fatigue. However, in the AB-group, performance was below chance for the smallest offsets. This may represent anterograde interference and suggests that subjects were processing the aligned stimuli (for the smallest offsets) as being offset to the side consistent with the A training.

## Discussion

Our results confirm that interference of learning on task A can occur if a subsequent task B is performed immediately after task A. The control group performing only task A showed a significant improvement after training. Moreover, a group run without the eye-tracker showed no retrograde disruption of task B on task A, similar to the findings of Aberg and Herzog [12]. These results suggest that visual perceptual learning of peripheral 3-dot hyperacuity can suffer from retrograde interference when subjects' eye movements are controlled.

To address why the AB training with and without the eye-tracker gives rise to two opposite outcomes, one must be considered the stimuli presented in the hyperacuity task. The three-dot stimuli were constantly presented in the lower-right visual field, a location that was highly predictable. These stimuli were presented on a mostly blank screen, other than the fixation point, and the sudden onset of the 3-dots can serve to draw eye-movements. In addition, unlike other studies of perceptual learning, such as the classic texture discrimination task [21], there was no central task to facilitate subjects' fixation. Therefore, it is difficult for subjects to maintain fixation throughout the experiment without an aid. While subjects in the gaze-free group were told to keep tight fixation on the central cross throughout the experiment, a number of subjects expressed that they tried their best, but that they inadvertently made occasional eye-moments towards the target stimuli. In these cases, when subjects foveated the target, the stimuli would be straightforward to discriminate, even for the hardest condition. Accordingly, two subjects, who were dropped after first day, exhibited evenly high accuracy (around 90%) among different offsets (data not shown).

So, why was learning found in the gaze-free group of the present study, and in Aberg and Herzog [12], but not in Seitz et al [7], when none of these experiments employed an eye-tracker? A hint that eye-movements may have occurred in all of these studies is that the gaze-free group, and both Seitz et al [7] and Aberg and Herzog [12], showed above chance performance in the hardest conditions, whereas this was not observed in the fixation conditions of the present study (even for the A-only group). While it is difficult to speculate regarding the extent to which subjects in Seitz et al [7] and Aberg and Herzog [12] did or did not maintain fixation, we suspect that at least some of the differences found between these studies may be due to the extent to which subjects maintained fixation during those experiments. We thus postulate that subjects in Seitz et al [7] were better at maintaining fixation. However, this speculation cannot be proven given that no eye-tracking data exists from those experiments. While it could be interesting to perform a new experiment to address our claim that eye-movement strategies change during the learning process, the primary goal of the present manuscript was to see if results of retrograde interference could be replicated under controlled conditions, which we have done.

It is difficult to determine precisely the key factors that contributed to possible eye movement differences, and otherwise, to the divergent findings across these experiments, however, there were a number of differences between our studies and that of Aberg and Herzog [12]. For example, instructions were different in Aberg and Herzog [12], in that they explicitly informed subjects of the offset side in each condition, whereas we did not. Furthermore, their stimuli looked qualitatively different in that there appeared to be some apparent motion for the central dot between the two presentation intervals, whereas this was not observable in our experiment (Seitz's personal observations). In Seitz et al [7] and Aberg and Herzog [12] breaks were given every 20 trials, whereas in the current study breaks were given every 100 trials. Also, our present study and Seitz et al [7] employed chinrest/forehead restraints, however, one was not used by Aberg and Herzog [12]. Furthermore, different subject populations were used and different experimental equipment was employed. How these factors, and the numerous other experimental differences that are unaccounted for, play a role in the observed findings remains a target of further research.

Given that even an occasional lapse in fixation can cause a large difference in observed results, we suggest that it is imperative to control subjects' eye movements in visual perceptual learning tasks that involve predictable presentation of peripheral stimuli. In our study, the eye-tracker ensured that subjects strictly performed fixation at the central cross when stimuli were presented on periphery; the trial wouldn't start if subjects didn't fixate at the center, and any eye-movement deviating away the fixation cross during stimuli presentation was caught by eye-tracker and the trial was skipped. Under the control of eye-tracker, we assume the chance of subject cheating in this experiment has been reduced to minimum, and the entire task was performed exactly on subject's peripheral vision instead of foveal vision. It is important to note, that the lack of eye-movement control in out gaze-free group provides ambiguity regarding the true nature of the learning in that study. It may be that case that retrograde interference occurred but we failed to observe it due to contamination from eye-movements. Further research is warranted to determine whether eye-movements in the gaze-free group actually prevented retrograde interference from occurring or merely reduced our ability to detect such interference.

Furthermore, it is important to note that the general observation by Aberg and Herzog that retrograde interference is not ubiquitous in perceptual learning is not called into question by our findings that retrograde interference can occur in perceptual learning. Notably, we only dispute the conclusion regarding one of the five studies included in the Aberg and Herzog [12] paper. Their other studies were run in central vision and are unlikely to have been impacted by subjects' eye-movements. While a variety of perceptual learning paradigms do demonstrate signs of retrograde interference [7], [8], [9], [10], [11], Aberg and Herzog's study make clear that retrograde interference is not ubiquitous in perceptual learning.

In conclusion, we suggest that retrograde interference is a common process across studies in perceptual learning [7], [8], [9], [10], [11], [22] and that it may share processes with retrograde interference in reading [1] and motor learning tasks [2], [3], [4], [5], [6]. However, retrograde interference may not be ubiquitous [12] and it certainly depends upon the details of the training task. Future research is definitely needed to gain a greater understanding of the processes that lead to interference of perceptual learning.

Furthermore, we conclude that taking advantage of eye-tracking technologies to not only track, but also to control for, eye-movements can provide needed clarity in studies of perceptual learning, particularly those involving presentation of stimuli in the visual periphery. Eye-tracking in peripheral perceptual tasks is very important because eye-movements, even on a small percentage of trials, can turn a difficult peripheral task into an easy foveal task. These occasional lapses can have a profound effect on measures of performance that emulate sensitivity changes and confound results. While the impact of eye-movements will have a greater impact in some studies (in particular studies employing peripheral tasks) than others, it is likely that eye-movements played a role in a large number of studies reported in the literature, including both Seitz et al [7] and Aberg and Herzog [12], and that without being measured and controlled for, readers are left guessing how they impacted the results of those studies.

## References

[1] Müller GE, Pilzecker A (1900). Experimentelle Beiträge zur Lehre vom Gedächtnis.. Z Psychol Ergänzungsband.

[2] Walker MP, Brakefield T, Hobson JA, Stickgold R (2003). Dissociable stages of human memory consolidation and reconsolidation.. Nature.

[3] Brashers-Krug T, Shadmehr R, Bizzi E (1996). Consolidation in human motor memory.. Nature.

[4] Shadmehr R, Brashers-Krug T (1997). Functional stages in the formation of human long-term motor memory.. J Neurosci.

[5] Caithness G, Osu R, Bays P, Chase H, Klassen J (2004). Failure to consolidate the consolidation theory of learning for sensorimotor adaptation tasks.. J Neurosci.

[6] Osu R, Hirai S, Yoshioka T, Kawato M (2004). Random presentation enables subjects to adapt to two opposing forces on the hand.. Nat Neurosci.

[7] Seitz AR, Yamagishi N, Werner B, Goda N, Kawato M (2005). Task-specific disruption of perceptual learning.. Proc Natl Acad Sci U S A.

[8] Sasaki Y, Yotsumoto Y, Chang LH, Watanabe T (2009). Interference and feature specificity in visual perceptual learning.. Vision Research.

[9] Mednick S (2010). REM sleep prevents interference in the texture discrimination task.. Journal of vIsion.

[10] Been M, Jans B, Lowet E, Arnoldussen D, De Weerd P (2010). Visual interference by a second learning experience is strongest during asymptotic learning.. Perception.

[11] Petrov AA, Dosher BA, Lu ZL (2005). The dynamics of perceptual learning: An incremental reweighting model.. Psychological Review.

[12] Aberg KC, Herzog MH (2010). Does Perceptual Learning Suffer from Retrograde Interference?. PLoS ONE.

[13] Tartaglia EM, Aberg KC, Herzog MH (2009). Perceptual learning and roving: Stimulus types and overlapping neural populations.. Vision Research.

[14] Otto TU, Herzog MH, Fahle M, Zhaoping L (2006). Perceptual learning with spatial uncertainties.. Vision Research.

[15] Kuai SG, Zhang JY, Klein SA, Levi DM, Yu C (2005). The essential role of stimulus temporal patterning in enabling perceptual learning.. Nat Neurosci.

[16] Yu C, Klein SA, Levi DM (2004). Perceptual learning in contrast discrimination and the (minimal) role of context.. J Vis.

[17] Zhang JY, Kuai SG, Xiao LQ, Klein SA, Levi DM (2008). Stimulus coding rules for perceptual learning.. PLoS Biol.

[18] Frey U, Huang YY, Kandel ER (1993). Effects of cAMP simulate a late stage of LTP in hippocampal CA1 neurons.. Science.

[19] Brainard DH (1997). The Psychophysics Toolbox.. Spat Vis.

[20] Pelli DG (1997). The VideoToolbox software for visual psychophysics: Transforming numbers into movies.. Spatial Vision.

[21] Karni A, Sagi D (1991). Where practice makes perfect in texture discrimination: evidence for primary visual cortex plasticity.. Proc Natl Acad Sci U S A.

[22] Sotiropoulos G, Seitz AR, Series P (2011). Perceptual learning in visual hyperacuity: A reweighting model.. Vision Res.

